# Αlpha 2a-Adrenoceptor Gene Expression and Early Life Stress-Mediated Propensity to Alcohol Drinking in Outbred Rats 

**DOI:** 10.3390/ijerph120707154

**Published:** 2015-06-25

**Authors:** Erika Comasco, Aniruddha Todkar, Linnea Granholm, Kent W. Nilsson, Ingrid Nylander

**Affiliations:** 1 Department of Neuroscience, Uppsala University, 75124 Uppsala, Sweden; E-Mail: aniruddha.todkar@neuro.uu.se; 2 Neuropharmacology, Addiction & Behaviour Group, Department of Pharmaceutical Biosciences, Uppsala University, 75124 Uppsala, Sweden; E-Mail: linnea.granholm@farmbio.uu.se; 3 Centre for Clinical Research, Västerås Central Hospital, 72189 Västerås, Sweden; E-Mail: kent.nilsson@ltv.se

**Keywords:** α2A-adrenoceptor, alcohol, brain, gene expression, rat, maternal separation, stress

## Abstract

Stressful events early in life, later high alcohol consumption and vulnerability to alcohol use disorder (AUD) are tightly linked. Norepinephrine is highly involved in the stress response and the α2A-adrenoceptor, which is an important regulator of norepinephrine signalling, is a putative target in pharmacotherapy of AUD. The aim of the present study was to investigate the effects of early-life stress and adult voluntary alcohol drinking on the α2A-adrenoceptor. The relative expression and promoter DNA methylation of the *Adra2a* gene were measured in the hypothalamus, a key brain region in stress regulation. A well-characterized animal model of early-life stress was used in combination with an episodic voluntary drinking in adulthood. Alcohol drinking rats with a history of early-life stress had lower *Adra2a* expression than drinking rats not exposed to stress. Alcohol intake and *Adra2a* gene expression were negatively correlated in high-drinking animals, which were predominantly rats subjected to early-life stress. The results provide support for a link between early-life stress, susceptibility for high alcohol consumption, and low *Adra2a* expression in the hypothalamus. These findings can increase our understanding of the neurobiological basis for vulnerability to initiate risk alcohol consumption and individual differences in the response to α2A-adrenoceptor agonists.

## 1. Introduction

Preclinical and clinical studies provide strong evidence of a link between stressful life events and high alcohol consumption, vulnerability to alcohol use disorder and relapse [1]. Translational approaches point to critical periods of development, such as the first postnatal weeks in rodents, that corresponds to infancy and early childhood in humans [2,3], where environmental impact is highly influential in shaping the brain and behaviour [4,5]. Environmental stressors during these sensitive time windows interact with the genetic make-up and influence brain neuroplasticity and adaptive capability in the long-term which are of relevance to both risk and resilience to develop AUD [6]. As reviewed in [7,8,9,10], substantial evidence has been provided for an effect of early life stress on alcohol consumption in adulthood in rodents.

A number of central and peripheral biological systems are activated in response to stress and it is a challenge to scrutinize their association with alcohol consumption and AUD. One candidate neurotransmitter is norepinephrine that is involved in the peripheral as well as the hypothalamic pituitary adrenal (HPA) response to stress [1,11]. Stress activates norepinephrine neurons and previous exposure to stressors sensitizes later stress-induced norepinephrine responses [11]. Norepinephrine is also implicated in alcohol consumption and in stress-induced reinstatement of alcohol seeking [12]. The α2A-adrenoceptor is an important regulator of norepinephrine signalling and an putative pharmacological target. Manipulations of the α2A-adrenoceptor have been shown to affect alcohol intake; yohimbine, an α2A-adrenoceptor antagonist, increases norepinephrine release, induces stress- and anxiety-like responses, and reinstates alcohol seeking after extinction [13,14]. On the other hand, α2A-adrenoceptor agonists decrease availability of norepinephrine in the synaptic cleft and reduce alcohol consumption [15,16], as well as alcohol deprivation effect, alcohol seeking behaviour, and cue/priming-induced reinstatement in high drinking rats [16]. These facts, together with the findings of an association between polymorphisms in the *Adra2a* gene and positive family history of AUD [17], call for molecular studies of the link between the α2A-adrenoceptor, stress and alcohol drinking.

The present study examines the putative link between stress, alcohol drinking and the α2A-adrenoceptor by investigating *Adra2a* gene expression in voluntary drinking adult rats with or without exposure to early-life stress. The hypothesis was that early-life stress conditions associated with greater propensity for high alcohol consumption later in life will induce long-term down-regulation of *Adra2a* expression, and changes in DNA methylation, in the hypothalamus. Gene expression and promoter DNA methylation of the *Adra2a* gene were analysed in the hypothalamus, an important integrative area in stress regulation, and a terminal region for norepinephrine projections [18]. 

## 2. Experimental Section 

### 2.1. Animals

Time mated Wistar dams (n = 25; RccHan:WI, Harlan, Europe) arrived at gestation day 15. This is the least sensitive phase during pregnancy and was chosen to minimize the influence of stress related to travel. No signs of negative impact of the transport were noticed during acclimatization in the animal facility and the delivery was normal in all females. After birth (postnatal day (PND) 0) the pups were sexed and cross-fostered to avoid the use of biological littermates in the same experimental groups. Each litter contained 10 pups (six males and four females), and the litters were randomly assigned to the different experimental groups. Only males were used in the present study. The study was approved by the Uppsala Animal Ethical Committee (C32/11) and followed the guidelines of the Swedish Legislation on Animal Experimentation (Animal Welfare Act SFS1998:56) and the European Communities Council Directive (86/609/EEC).

**Figure 1:**
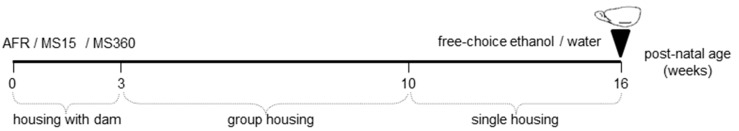
Experimental outline. The rats were subjected to animal facility rearing (AFR), 15 min (MS15) or 360 min (MS360) of maternal separation during the first three postnatal weeks. The animals were group housed during adolescence and single housed during ethanol/water consumption. One additional AFR group was included with water-drinking AFR rats that were group-housed throughout the experiment.

### 2.2. Early-Life Rearing Conditions

A rodent maternal separation (MS) model was used to simulate different early life conditions during the first three postnatal weeks (Figure 1). Based on previous studies, prolonged daily MS (360 min; MS360) was used to simulate a risk environment [7] and short MS (15 min; MS15) was used as control to MS360. The separations were performed during the light period and started at 9 AM. The MS procedure has been described in detail elsewhere [19]. The litters were weighed on PND 0, 3, 7, 10, 13, 16 and the cages were changed on PND 7 and 16. The separations were always performed in the same animal rooms and only one person performed all separations and care taking. Animal facility reared (AFR) rats were included in the study for assessment of single housing and ethanol drinking in rats subjected to conventional laboratory rearing conditions. The animals in the AFR group were left undisturbed with the exception of cage change (PND 7, 16) and weighing of the litter (PND 0, 7, 16). On PND 22 all animals were weaned and then group housed, three per cage, during adolescence. 

### 2.3. Voluntary Ethanol Consumption

On postnatal week 10, the MS rats were randomly assigned to either water-drinking (MS15W, n = 10; MS360W, n = 10) or ethanol-drinking groups (MS15E, n = 10; MS360E, n = 20). Twice as many rats were included in the MS360 group based on previous findings of subgroups with responder and non-responder rats regarding ethanol intake [7]. The AFR rats were assigned to water (AFRW, n = 9) and ethanol drinking (AFRE, n = 11), respectively. The rats were single housed for individual fluid measurements until decapitation at week 16. However, one additional group of AFR rats (n = 7) were group housed during week 10 to 16 to assess the housing effect.

The rats exposed to ethanol had access to non-sweetened ethanol (5% or 20% made from Etanol 96%; Solveco AB, Rosersberg, Sweden) and water in a two-bottle free choice paradigm for three consecutive days with four drug-free days in-between. The first week the rats had free access to 5% ethanol for 24 h and the next week limited access to 5% for 2 h; the following five weeks they had access to 20% ethanol in 2 h sessions for the three days. This drinking paradigm is developed to mimic human episodic drinking patterns in habitual drinking, with repeated drinking days and non-drinking days in-between [20,21]. Various intermittent models are commonly used in voluntary drinking models to increase the ethanol intake, and intermittent drug exposure with drug-free days in-between has also been shown to be necessary to induce neurobiological alterations similar to those seen in the transition to habitual and compulsive drinking [22]. The limited access restricted to 2 h is a better choice to ensure less variation in biological parameters due to individual differences in drinking bouts in a 24 h access paradigm. Ethanol and water were changed every session and the bottle position was altered every day to avoid position preference. Bottles with nipples were employed to minimize spillage. At the end of each session, the ethanol and water intake was quantified by weighing the bottles. Care was taken to minimize spillage. At postnatal week 16, the rats were decapitated. The ethanol intake is reported in the supplementary material. The ethanol-drinking animals were sacrificed immediately after a 2 h drinking session. The hypothalamus was removed from the brain and immediately frozen on dry ice and stored at −80 °C. 

### 2.4. Adra2a Gene Expression Analyses

*RNA isolation:* RNA was isolated from rat hypothalamus using AllPrep DNA/RNA/miRNA Universal Kit according to the manufacturer’s protocol (Qiagen AB Sollentuna, Sweden). Quantification of the nucleic acid was carried out using a Nanodrop ND 1000 spectrometer. 

*cDNA synthesis*: RNA (700 ng) was converted to cDNA using the QuantiTect Reverse Transcription Kit (Qiagen AB Sollentuna, Sweden). The manufacturer’s protocol was followed including a genomic DNA (gDNA) wipe-out reaction. The final cDNA synthesis reaction was performed at 42 °C for 35 minutes, and the reaction was inactivated at 95 °C for 5 minutes. The newly synthesized cDNA was diluted 20 times and stored at −20 °C. 

*Gene expression analyses:* The newly synthesized cDNA was diluted 20 fold was used to assess the expression of *Adra2a* as well as *Actb, Gapdh* and *Rpl19,* as housekeeping genes, using CFX96 Touch Real-Time PCR Detection System real time PCR. Primers were designed using Primer 3 (http://frodo.wi.mit.edu/) and cross-checked using Primer Map (http://www.bioinformatics.org/sms2/primer_map.html) (Table S1). The final reaction mixture of 20 µL contained iQ SYBR Green Supermix (Biorad Sweden) and 0.15 µM of each primer, and 3 µL cDNA template; and each sample was run in triplicates. The PCR conditions are listed in Table S1. A three-step control was performed to assess gDNA contamination: (1) On column DNase treatment during the extraction process; (2) gDNA wipe-out reaction prior to cDNA synthesis. Moreover, each real time PCR plate contained samples belonging to all experimental groups. 

*Data analysis:* Data of the relative fluorescence unit (RFU) were collected, and the PCR efficiency and corrected c_q_ values, adjusted for mean threshold and PCR efficiency across the plates, were calculated using the LinregPCR open source software [23]. Samples with normalized Cq values that had a standard deviation of more than 0.5 were excluded. Relative gene transcripts levels were determined using the ∆CT method (BioRad real time PCR application guide, Bio-Rad, #170-9799). All the laboratory and preprocessing analyses were performed in a blind manner. 

### 2.5. Adra2a Promoter DNA Methylation Analyses

The most proximal CpG island to the transcription start site of the *Adra2a* gene was targeted to assess DNA methylation patterns at 19 CpG sites using quantitative pyrosequencing. A detailed description of the method used is provided in the supplementary material.

### 2.6. Statistics

One outlier belonging to the water-drinking, group-housed AFR group had relative gene expression data >2.5 standardized score and was excluded. Comparisons of *Adra2a* gene expression between groups were analysed with one-way ANOVA test and Fisher *post-hoc* test. General Linear Model two-way ANOVA tests were used to examine main effects of stress and ethanol as well as interaction between stress and ethanol. Correlation between *Adra2a* gene expression and ethanol consumption was analyzed with the Spearman Rank correlation test. 

## 3. Results 

### 3.1. Ethanol Intake and Preference

**Figure 2:**
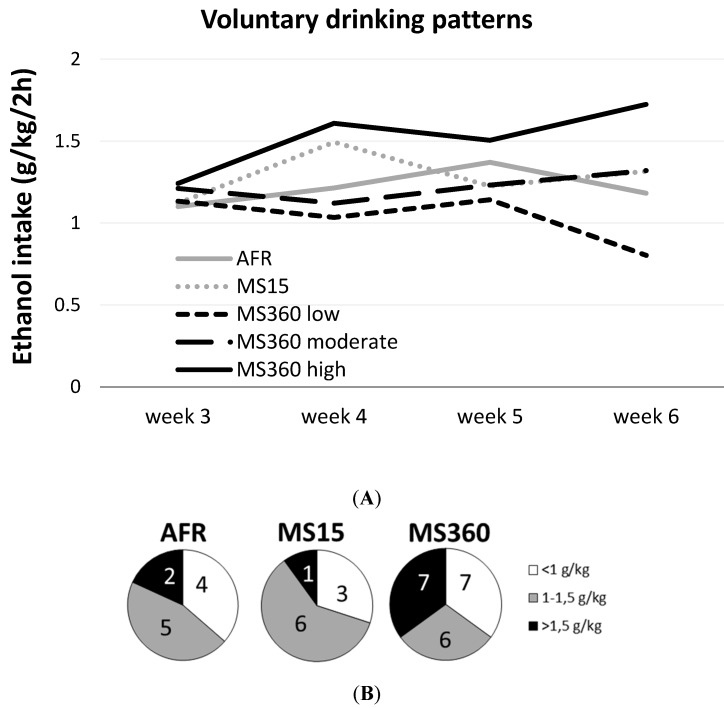
(**A**) The weekly voluntary ethanol consumption patterns during the four weeks with free access to 20% ethanol three days a week in 2 h sessions. The figure shows the drinking patterns in the MS15 and AFR groups and in the three subgroups of MS360 rats with high (>1.5 g/kg/2h), moderate (1–1.5 g/kg/2h) and low (<1 g/kg/2h) ethanol intake during week 6. (**B**) The number of rats drinking >1.5, 1–1.5 and <1 g/kg/2h, in the AFR, MS15 and MS360 groups, respectively. MS360: 360 min maternal separation.

The consumption of 20% ethanol was stable over time in the AFR and MS15 rats as evidenced by the high correlation between ethanol week three and six (AFR, r = 0.768, *p* = 0.015, and MS15, r = 0.870, *p* = 0.009) whereas the rats within the MS360 group displayed a heterogeneous pattern and no correlation (r = 0.147, *p* = 0.521). Based on the ethanol intake at week six the MS360 rats were subgrouped into high (>1.5 g/kg/2h), moderate (1–1.5 g/kg/2h) or low (<1 g/kg/2h) drinkers. Different drinking patterns were revealed in these subgroups; the high drinking rats increased their ethanol consumption over time whereas the moderate drinkers had a stable intake pattern and the low drinking rats had a decreased intake (Figure 2A). 

The change over time differed between these groups (H = 6.12; *p* = 0.047) with a significant difference between the high and low drinking MS360 rats (Z = 2.17; *p* = 0.030). In the MS360 group, seven rats (35%) consumed > 1.5 g/kg/2h as compared to one MS15 rat (10%) and two AFR rats (18%) (Figure 2B). Comparing the ethanol intake and preference during the last week before decapitation in the AFR, MS15 and the entire MS360 group revealed no statistically significant differences. The ethanol intake, median (min–max), in the different groups was: AFR, 1.18 (0.62–1.66); MS15, 1.32 (0.39–1.77); MS360, 1.32 (0.6–2.05). The ethanol preference median (min–max) was: AFR, 62.2 (42.9–86.2); MS15, 63.8 (26.2–87.7); MS360, 75.4 (22.0–90.6) (gr/kg). The weekly median ethanol consumption including min-max values during all weeks is shown in Table S2.

### 3.2. Adra2a Gene Expression 

**Figure 3:**
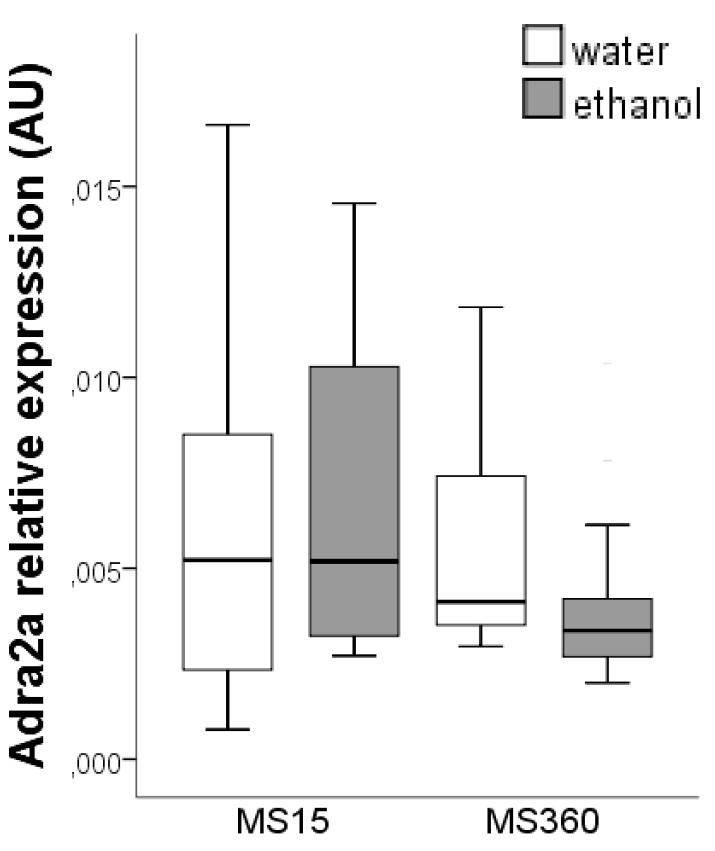
*Adra2a* gene expression in the hypothalamus of water- and ethanol-drinking MS15 and MS360 rats. MS: maternal separation.

Firstly, we examined whether early-life stress (MS360 *versus* MS15) causes changes in Adra2a gene expression and results in different ethanol-induced effects in adulthood. A two-factor analysis comparing water- and ethanol-drinking MS15 and MS360 rats revealed that there was a trend in the main effect of stress (*F* = 3.13; *p* = 0.083), no statistically significant main effect of alcohol (*F* = 0.03; *p* = 0.854) and no interaction between stress and ethanol (*F* = 2.25; *p* = 0.141) (Figure 3). 

**Figure 4:**
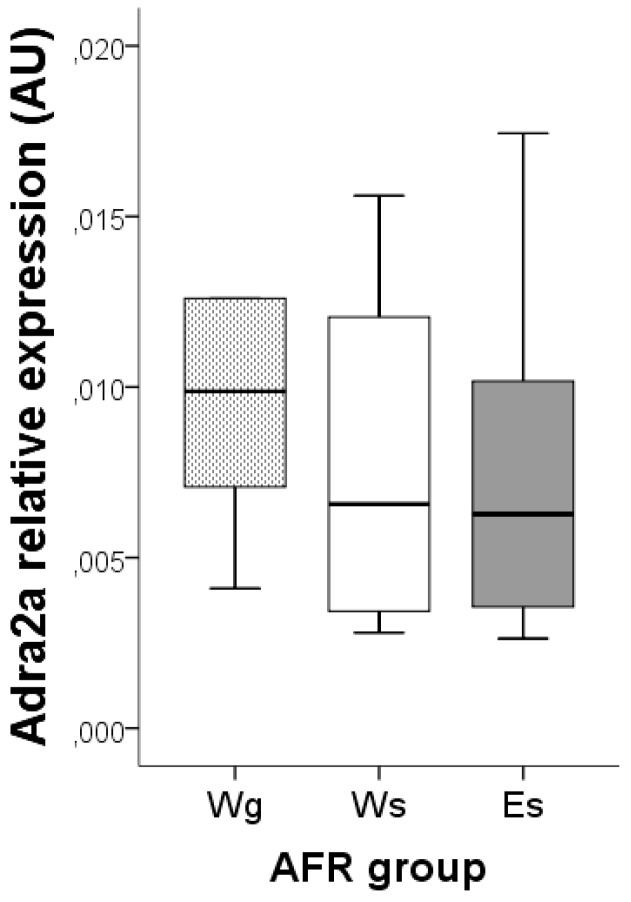
*Adra2a* gene expression in the hypothalamus of AFR adult rats subjected in adulthood to single-housing (s), and given a free choice between ethanol (E) and water or water only (W). One group of rats were group-housed also in adulthood (AFRWg). AFR: animal facility reared; E: ethanol; g: group housed; s: single housed; W: water.

Secondly, we examined the effect of voluntary drinking on *Adra2a* gene expression in rats subjected to conventional animal facility rearing (AFR) conditions. Single- and group-housed AFR rats were used as controls to exclude possible confounding by single housing stress. There was no statistical significant difference in gene expression between the three AFR groups (*F* (2, 25) = 0.87; *p* = 0.432). That is, neither single housing nor ethanol drinking in adult rats affected *Adra2a* gene expression (Figure 4). 

**Figure 5:**
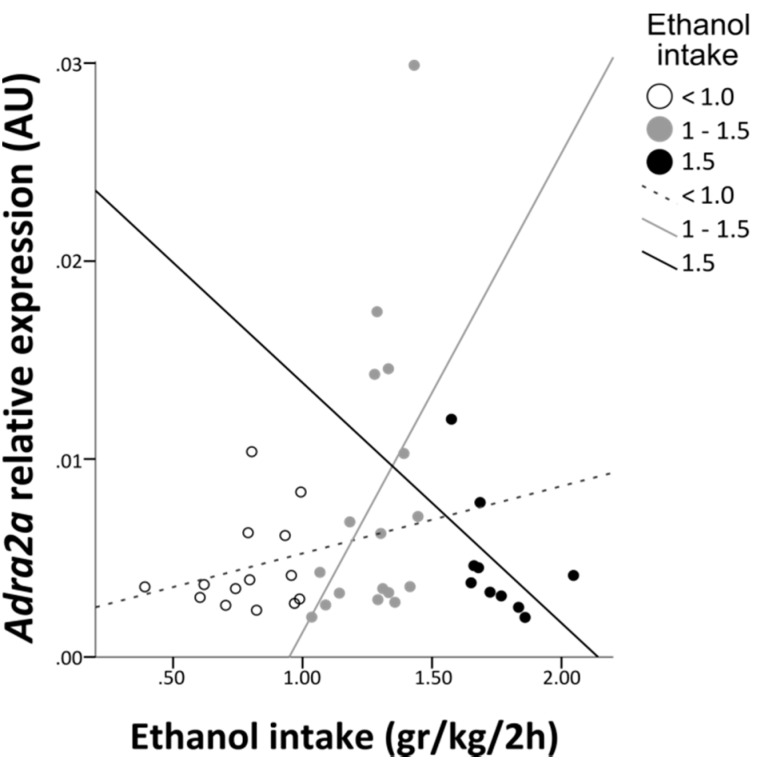
The relationship between *Adra2a* gene expression in the hypothalamus and ethanol intake in drinking rats divided into three subgroups with high > 1.5, moderate 1–1.5 and low < 1 g/kg/2h ethanol consumption, respectively.

Thirdly, based on different effects of a α2A-adrenoceptor agonist in high and low-drinking rats [16], the individual *Adra2a* expression and ethanol intake was examined in all ethanol-drinking rats (n = 41). The results revealed different responses in animals with high, moderate and low ethanol consumption. The correlation analysis of ethanol consumption and *Adra2a* gene expression in all rats drinking > 1.5 g/kg/2h (n = 10) revealed a negative correlation between ethanol intake and *Adra2a* gene expression (*r* = ‒0.673; *p* = 0.044) (Figure 5). Most of these high-drinking rats were MS360 rats (70%). In contrast, there was a trend towards a positive, but not statistically significant, correlation (*r* = 0.431; *p* = 0.085) in the moderate drinking rats (n = 17) and no significant correlation in the low drinking rats (n = 14) (Figure 5). Furthermore, the ethanol-drinking MS360 rats had the lowest *Adra2a* gene expression (F (2, 38) = 3.37; *p* = 0.045); MS360 rats had lower expression than the MS15 rats (*p* = 0.03) and a trend to lower than the AFR rats (*p* = 0.066) whereas no differences (*p* = 0.667) were seen between MS15 and AFR rats.

Lastly, we examined whether DNA methylation at the *Adra2a* promoter region is a mediating factor of ethanol-induced effects. The promoter region at the CpG island most proximal to the transcription start site was mainly un-methylated in all samples, thus no statistics were performed (Supplementary Material).

## 4. Discussion 

The present study employed a voluntary drinking design in combination with a well-characterized animal model for early-life stress to examine the link between stress, alcohol and *Adra2a* gene expression in the hypothalamus. The main findings were lower *Adra2a* gene expression in alcohol drinking rats exposed to early-life stress compared to other drinking rats, predominance of rats subjected to early-life stress amongst the high-drinking animals, and a negative correlation between alcohol intake and *Adra2a* gene expression solely in the high-drinking animals.

It has been proposed that genetic and environmental factors interact in promoting AUD via the response to stress [24]. Risk is conferred by stress in childhood and adolescence, such as maltreatment and other forms of poor parenting, parental mental disorders, inter-parental violence, low socio-economic status, and negative peer influences [25,26]. These stressors modify brain development during critical periods [5,27]. Early environmental stressors, such as poor maternal care in rodents and human children maltreatment, have long-term effects on the stress response, as well as on mental health, learning, memory, and neuroplasticity [25,28,29]. Thus, associations between childhood physical abuse and subsequent alcohol use and AUD may be driven, at least in part, by interactions with genes related to stress reactivity [24,30,31,32]. However, there is not a straight forward link between environmental stress and alcohol consumption. Other factors like access [33] or exposure to alcohol [34], reasons for drinking [35] and individual alcohol preferences [36] modulates the actual consumption. Moreover, results also indicate that the amount of alcohol consumption is associated to different levels of gene expression [34]. 

Experimental animal studies allow thorough examination of the brain under controlled environmental conditions and provide valuable insight into the mechanisms underlying early-life impact on brain and behaviour [5,37]. For example, maternal care in rodents influences stress response, neuroplasticity and has long-term repercussions on behaviour in later stages of life of the offspring [37], likely through changes in the DNA methylation make-up of genes involved in HPA axis function [38]. Disturbed social interactions between the dam and her pups are known to induce long-term neurobiological changes and also affect the propensity to voluntarily drink alcohol in adulthood [7,8]. Therefore the aim of the present study was to investigate the combined effects of stress [7] and different levels of alcohol consumption [39] on *Adra2a* gene expression. 

A well-characterized maternal separation model was used to disturb the social interactions between dam and pups. Prolonged (e.g., MS180 or MS360) and shorter (e.g., MS15) maternal separation during the first three postnatal weeks is associated with risk and protection, respectively, in relation to alcohol drinking behaviour and alcohol-induced effects in adulthood [7,8,9]. Stress during a vulnerable period of development, simulated herein by MS360 during PND 1-21, was expected to be associated with lower *Adra2a* expression due to interference with critical steps in norepinephrine maturation and synaptogenesis. For example, the spontaneous firing rate of norepinephrine neurons in the locus coeruleus of rats peaks at PND 20, and the density of α2-adrenoceptors gradually increase during the postnatal period, with a peak at PND15 [40]. Stress, as well as *Adra2a* antagonists, causes increased norepinephrine in hypothalamus, and anxiety-like behaviour in rats and humans [14,41], and can contribute to vulnerability to high alcohol consumption. However, no statistically significant differences in *Adra2a* expression were seen between adult MS15 and MS360 rats in the present study. Importantly, the animals were group-housed with regular social contacts during adolescence but as young adults they were single housed and given free access to alcohol or used as water-drinking controls. Thus, the *Adra2a* expression was measured in single-housed animals and a possible effect on gene expression levels in group-housed animals cannot be excluded. Single housing is associated with stress [42], and since voluntary drinking designs often include single-housed animals, it is important to discriminate between a possible effect of housing and the effect of alcohol for any endpoint measured. This was tested in the AFR rats and the results indicated that *Adra2a* gene expression is not affected by single housing in adult animals. However, even though single housing had no effect in the AFR rats, it is not known how MS15 or MS360 would be affected. 

A limitation of the present study is the measurement of gene expression in the whole hypothalamus which is a heterogeneous brain structure, while techniques such as *in situ* hybridisation would have allowed investigating functional sub-nuclei. Moreover extra-hypothalamic brain regions should also be investigated. A strength is the use of outbred non-preferring rats, *i.e.*, not selectively bred for alcohol preference, that were allowed to freely drink on an intermittent three-day alcohol access paradigm with repeated periods of abstinence. This drinking pattern facilitates neurobiological changes observed during the transition from voluntary into compulsive alcohol drinking [22] and has the advantage over continuous drinking to mimic human habitual episodic drinking patterns. Furthermore, this design also allowed the examination of individual responses to alcohol and possible differences in *Adra2a* expression in animals that acquire higher or lower alcohol consumption when they have free access to alcohol. This is of interest based on recent results showing that the α2A-adrenoceptor agonist guanfacine is highly effective to reduce voluntary drinking in high-drinking rats but not in low-drinking rats [16]. All drinking animals were therefore divided into subgroups based on their voluntary alcohol intake behaviour. Interestingly, the correlation between *Adra2a* gene expression and alcohol intake differed in rats with higher or lower alcohol consumption, respectively. In the high-drinking rats, *i.e.*, those with consumption > 1.5 g/kg/2h week six, a negative correlation was found. This pattern was not seen in the moderate or low drinkers. The association between high alcohol consumption and low *Adra2a* expression found in the present study may contribute to explain the good response to treatment with α2A-adrenoceptor agonists in high-drinking animals [16].

On the basis of these results it may be hypothesized that individuals that are susceptible to acquire high alcohol consumption either respond with decreases in *Adra2a* expression when they drink or they have an inherent lower *Adra2a* expression and, as a consequence, higher synaptic availability of norepinephrine in the hypothalamus. Previous studies have shown that MS360 is a risk condition for high alcohol intake whereas MS15 rats have low alcohol consumption and do not increase intake over time [7,19]. Herein, using limited access to alcohol in 2 h sessions, no overall statistically significant difference was found in alcohol intake at the group level. A longer drinking period may be necessary to be able to observe group differences as seen in studies with continuous or intermittent 24 h access to alcohol [7]. However, in line with previous studies [7] a subgroup of MS360 rats (responders) was identified that increased alcohol intake over time and had higher alcohol intake towards the end of the drinking period. These MS360 rats constituted the majority of all rats with a consumption > 1.5 g/kg/2h and had the largest contribution to the negative correlation seen between alcohol intake and *Adra2a* expression. It was also shown that the alcohol-drinking MS360 rats had the lowest levels of *Adra2a* expression of all alcohol-drinking rats. These results indicate that the norepinephrine system is deranged in the MS360 responders, *i.e.*, individuals susceptible for high alcohol consumption after being exposed to early-life stress. These stress-induced changes are persistent into adulthood and become evident as altered responses to alcohol. 

## 5. Conclusions 

The present results provide support for a link between early-life stress mediated susceptibility for high alcohol consumption and low *Adra2a* expression in the hypothalamus. Rats subjected to early-life stress were highly represented among those rats that increased voluntary consumption over time and they had lower *Adra2* expression than other alcohol-drinking rats not exposed to stress. These findings can increase our understanding of the neurobiological basis for vulnerability to initiate risk alcohol consumption and give further clues about individual differences in the response to α2A-adrenoceptor agonists.

## Figures and Tables

**Figure S1:**
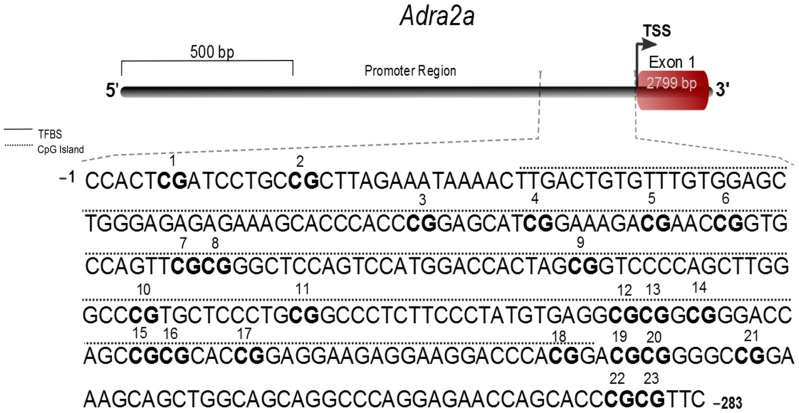
Promoter region of the *Adra2a* gene DNA sequence comprising the fragment (chr1:282178264-282178429; from −211 to −46 bases from TSS/ATG) and the location of the selected 19 CpG sites (3–21).

## Supplementary Material

### DNA Methylation Analysis

Target sequence: The promoter region of *Adra2a* was chosen to investigate potential DNA methylation patterns. The criteria used for selecting the target amplicons were the presence of Transcription Factor Binding Sites (TFBSs) and/or CpG islands in the promoter region, preferably close to the transcription Start Site. In addition, previous studies that investigated DNA methylation patterns of the genes of interest were scanned. Regarding the *Adra2a* gene, the selected amplicon was a 227 bp long region comprising a CpG island (Figure S1).

**Gene**	**Chromosome position**	**Position (ref to TSS)**	**Number of CpG Sites**
*Adra2a*	chr1: 282178264-82178429	−211 to −46	19

DNA isolation: DNA was isolated from rat brain tissue using the All Prep DNA/RNA QIAGEN kit. The DNA for analysing the *Adra2a* gene was extracted from the hypothalamus. Importantly, the DNA was isolated from the same cells clusters used for the messenger RNAs (mRNAs) isolation.

DNA methylation assay: Bisulphite quantitative pyrosequencing technique was used to assess the methylation pattern of the four genes using 500 ng of DNA at a concentration of 20ng/μL (EpigenDx (MA, USA)). DNA underwent Bisulphite Conversion, with an efficiency of more than 99%, using EZ DNA methylation kit (ZymoResearch, Inc., CA), thus converting un-methylated cytosine bases (C) into uracil bases (U). A. 0.2 μM of each primer with one of the PCR primers being biotinylated to purify the final PCR product using Sepharose beads. The PCR product was bound to Streptavidin Sepharose HP (Amersham Biosciences, Uppsala, Sweden), and the Sepharose beads containing the immobilized PCR product were purified, washed and denatured using 0.2 M NaOH solution and rewashed using the Pyrosequencing Vacuum Prep Tool (Pyrosequencing, Qiagen) as recommended by the manufacturer. Then 0.5 μM Pyrosequencing primer was annealed to the purified single-stranded PCR product. 10 μL of the PCR products were sequenced by Pyrosequencing PSQ96 HS System (Pyrosequencing, Qiagen), to detect the differentially methylated CpG sites, following the manufacturer’s instructions. To verify the efficiency of sodium bisulfite DNA conversion, each individual Pyrosequencing reaction included a non-CpG cytosine as an internal bisulfite modification control, while low, medium, and high methylated DNA samples were included as controls in each plate. Also, pyrosequencing used to do PCR bias testing for samples made of a mix of the unmethylated DNA control and in *vitro* methylated DNA at different ratios (0%, 5%, 10%, 25%, 50%, 75% and 100%) followed by bisulfite modification, PCR and pyrosequencing analysis. The percent methylation obtained from the mixing study showed high correlation with expected methylation percentages with a correlation coefficient of 0.96, indicating high quality methylation data.

Data analysis: Since the unmethylated CpG sites were converted into uracil (or Thymine), QCpG software, which is a pyrosequencing analysis software, generated a report showing the percentage of methylation by calculating the ratio between both C and T peaks in the pyrogram at the same position. The calculated percentage was graduated from 100%, indicating the highest methylation, to 0%, indicating not methylated site. There was no signal for one sample.

Statistical analyses: no statistics were performed as the majority of the sites was unmethylated (>98%).

**Table S1:** Primers for gene expression assessment with respective annealing temperatures.

Gene	Primers	Initial denaturation °C Time^#^	Denaturation °C Time^#^	Annealing °C Time^#^	Elongation °C Time^#^	Cycles	Melting curve
*Actb*	Forward: 5’CACTGCCGCATCCTCTTCCT 3’ Reverse: 5’ AACCGCTCATTGCCGATAGTG 3’	95 °C 3:00	95 °C 0:10	60 °C 0:30	72 °C 0:45	45	65 to 95 °C increment 0.5 °C
*Gapdh*	Forward: 5’ ACATGCCGCCTGGAGAAACCT 3’ Reverse: 5’ GCCCAGGATGCCCTTTAGTGG 3’	95 °C 3:00	95 °C 0:10	60 °C 0:30	72 °C 0:30	45	65 to 95 °C increment 0.5 °C
*Rpl19*	Forward: 5’CCAATGAAACCAACGAAATC 3’ Reverse: 5’TACCCTTCCTCTTCCCTA 3’	97 °C 3:00	95 °C 0:10	60 °C 0:30	72 °C 0:40	45	65 to 95 °C increment 0.5 °C
*Adra2a*	Forward: 5' GGTAAGGTGTGGTGCGAGAT 3’ Reverse: 5’ CAGCGCCCTTCTTCTCTATG 3’	95 °C 3:00	95 °C 0:10	60 °C 0:30	72 °C 0:45	49	65 to 95 °C increment 0.5 °C

^#^: minutes: seconds.

**Table S2:** The weekly voluntary ethanol consumption in AFR, MS15 and MS360 rats, and in the three subgroups of MS360 rats with high (>1.5 g/kg/2h), moderate (1–1.5 g/kg/2h) and low (<1 g/kg/2h) based on their ethanol intake during week six. The rats had access to ethanol three days a week in a two-bottle free choice between water and ethanol. The values represent the median ethanol intake, min–max. AFR, animal facility reared; MS15, 15 min maternal separation; MS360, 360 min maternal separation.

	5 %, 24h	5 %, 2h	20 %, 2h			
	Week 1	Week 2	Week 3	Week 4	Week 5	Week 6
**AFR**	1.61 0.29–4.16	0.40 0.18–0.75	1.10 0.50–2.11	1.21 0.59–1.42	1.37 0.77–2.08	1.18 0.62–1.66
**MS15**	1.89 0.87–3.40	0.67 0.32–0.99	1.12 0.75–1.61	1.49 0.89–2.13	1.23 1.05–1.74	1.32 0.39–1.77
**MS360**	1.53 0.21–3.28	0.57 0.09–1.14	1.20 0.29–1.98	1.11 0.56–2.15	1.28 0.38–2.52	1.32 0.60–2.05
**MS360 high**	1.13 0.22–2.74	0.65 0.25–1.14	1.24 0.94–1.98	1.61 0.80–2.15	1.51 0.38–1.9	1.72 1.65–2.05
**MS360 moderate**	1.95 0.56–3.28	0.62 0.09–0.8	1.21 0.29–1.6	1.12 0.56–1.45	1.23 0.75–2.01	1.32 1.04–1.41
**MS360 low**	1.43 0.21–1.75	0.57 0.34–0.73	1.13 0.48–1.85	1.03 0.87–1.35	1.14 0.71–2.52	0.80 0.60–0.99
